# Supplementary material for: Studies on the Drug Loading and Release Profiles of Degradable Chitosan-Based Multilayer Films for Anticancer Treatment
Source: Cancers (Basel). 2020 Mar 5;12(3):593. doi: 10.3390/cancers12030593 (PMC7140006; doi:10.3390/cancers12030593)
Supplement: Supplementary file 1 [file cancers-12-00593-s001.pdf]

Supplementary Materials

Studies on the Drug Loading and Release Profiles of Degradable Chitosan-Based Multilayer Films for Anticancer Treatment

Hyeongdeok Sun, Daheui Choi, Jiwoong Heo, Se Yong Jung and Jinkee Hong

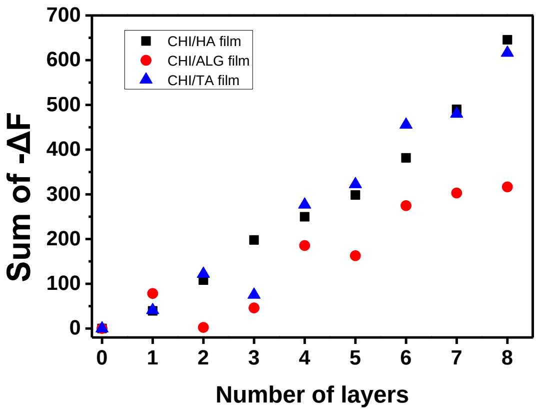

Figure S1. Stepwise frequency decrement of CHI/HA, CHI/ALG, and CHI/TA films as a number of layers measured by QCM.

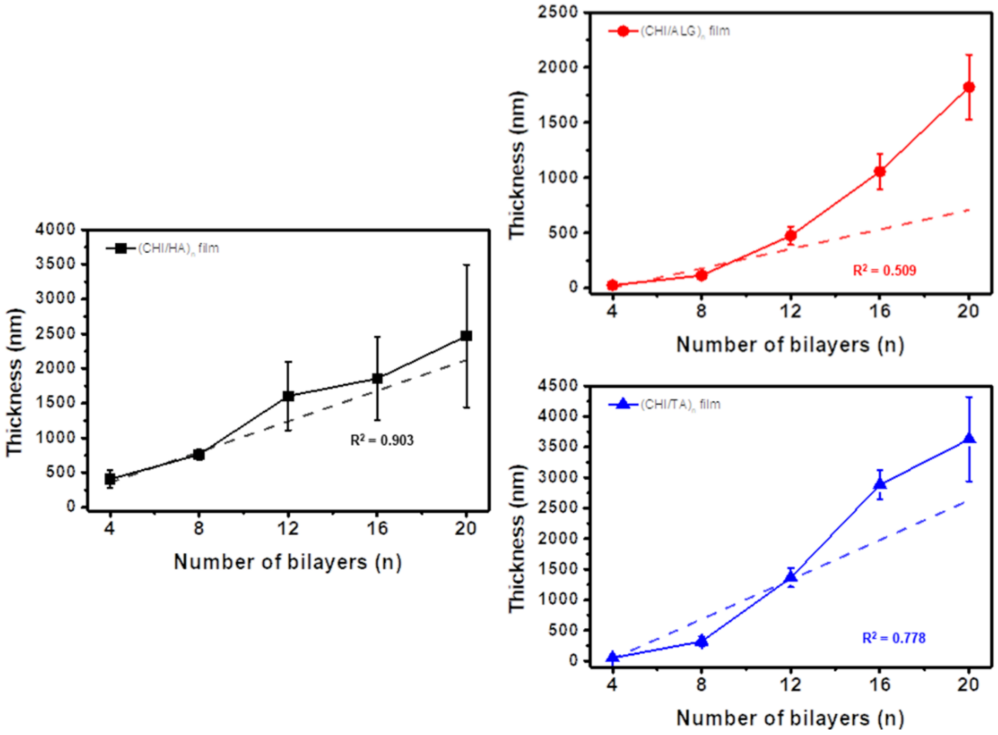

Figure S2. Multilayer films thickness growth with regression line as number of bilayers increased.

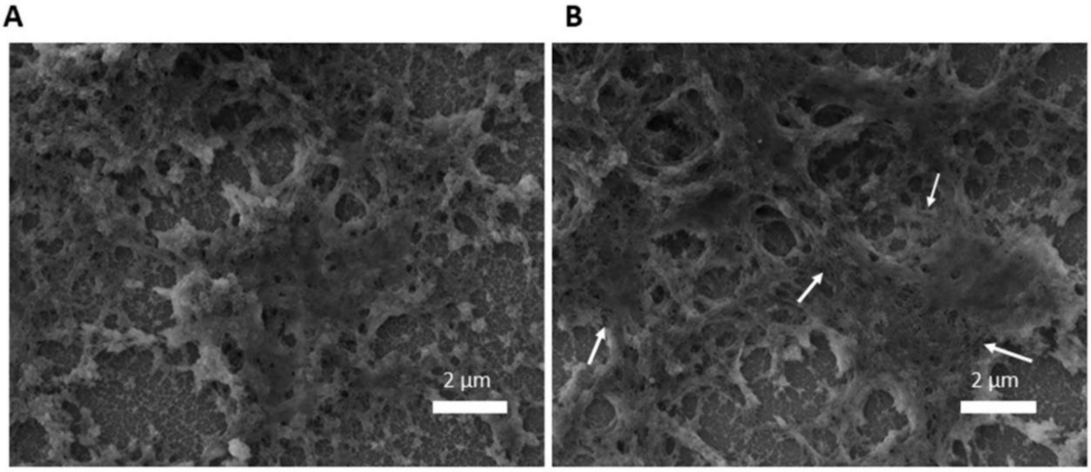

Figure S3. SEM images of CHI/TA film. (A) Bare CHI/TA film and (B) dipping in PBS buffer at 37 °C for 48 h. The white arrows indicate the dis-linked and disassembled CHI+TA aggregates in film.

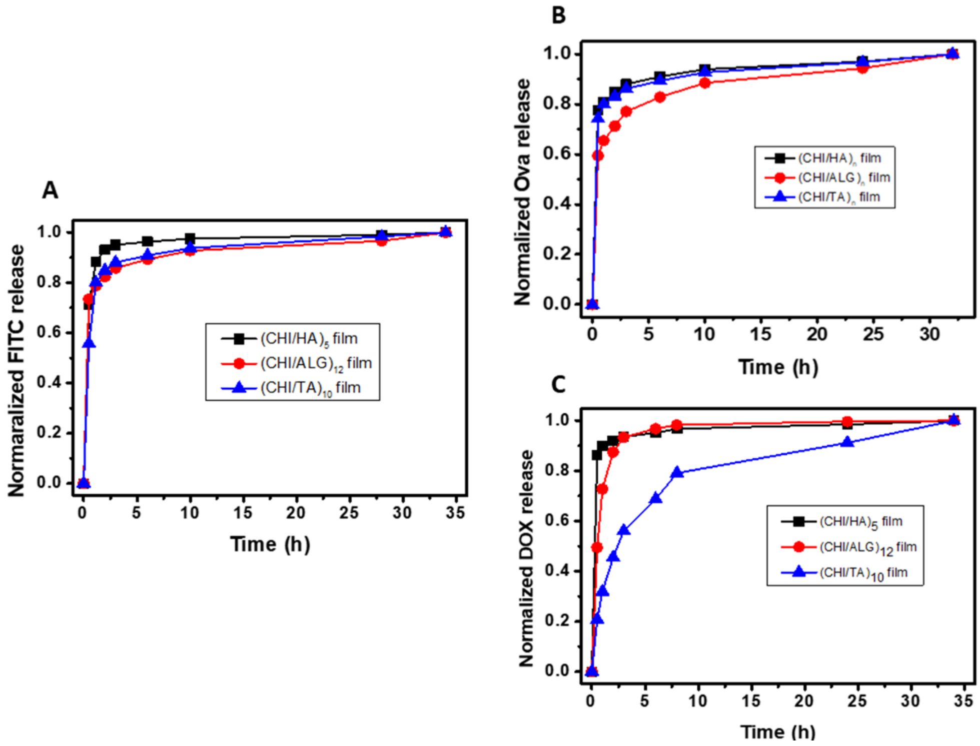

Figure S4. All release profiles of (A) FITC, (B) Ova, and (C) DOX, respectively.

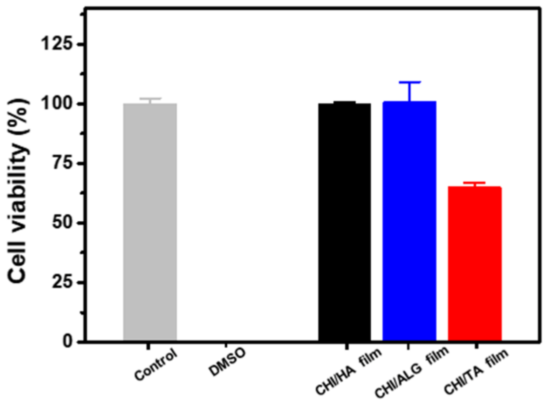

Figure S5. The cell viability of human dermal fibroblast cell.

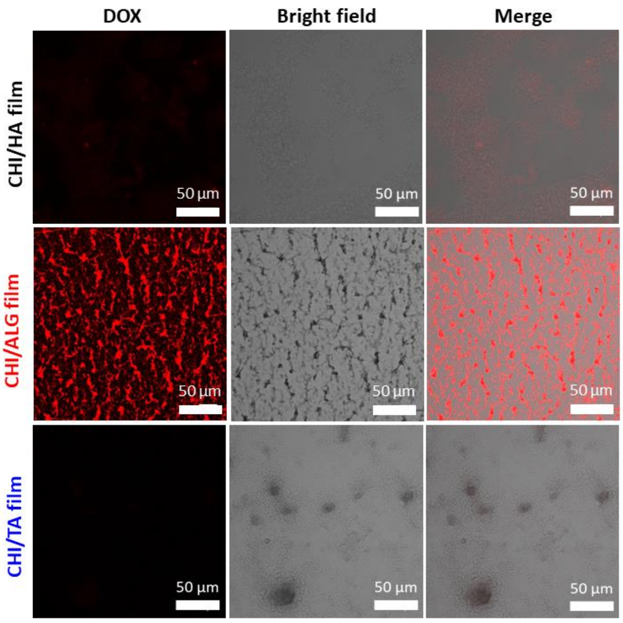

Figure S6. Confocal microscope image of DOX-incorporated multilayer films. Red fluorescence and bright field represent DOX and film structure.

Table S1. Table for degradation rate of multilayer films under 1× PBS at 37°C measured by changes in thickness.

Average Thickness (nm)	0 h	0.5 h	1 h	3 h	7 h	14 h	24 h	48 h
CHI/HA film	**475.89** ± 78.70	**292.21** ± 46.31	**247.66** ± 45.80	**244.24** ± 13.57	**238.60** ± 35.01	**226.71** ± 23.90	**221.71** ± 26.96	**234.34** ± 80.31
CHI/ALG film	702.26 ± 42.92	**274.85** ± 37.99	**257.61** ± 33.30	**251.32** ± 39.66	**242.70** ± 15.13	**284.02** ± 59.28	**279.34** ± 15.80	**264.90** ± 15.61
CHI/TA film	**1042.78** ± 179.54	**854.35** ± 153.16	**684.90** ± 97.87	**659.86** ± 205.16	**594.92** ± 92.29	**508.64** ± 53.29	**517.91** ± 91.40	**478.63** ± 82.43
